# Individual variation and the source-sink group dynamics of extra-group paternity in a social mammal

**DOI:** 10.1093/beheco/ary164

**Published:** 2019-01-14

**Authors:** Paula H Marjamäki, Hannah L Dugdale, Deborah A Dawson, Robbie A McDonald, Richard Delahay, Terry Burke, Alastair J Wilson

**Affiliations:** 1 Centre for Ecology and Conservation, University of Exeter, Penryn, Cornwall, UK; 2 NERC Biomolecular Analysis Facility, Department of Animal and Plant Sciences, University of Sheffield, Sheffield, UK; 3 School of Biology, Faculty of Biological Sciences, University of Leeds, Leeds, UK; 4 Environment and Sustainability Institute, University of Exeter, Penryn, Cornwall, UK; 5 National Wildlife Management Centre, Animal and Plant Health Agency, Gloucestershire, UK

**Keywords:** extra-group paternity, individual variation, *Meles meles*, parentage assignment, source-sink dynamics

## Abstract

Movement of individuals, or their genes, can influence eco-evolutionary processes in structured populations. We have limited understanding of the extent to which spatial behavior varies among groups and individuals within populations. Here, we use genetic pedigree reconstruction in a long-term study of European badgers (*Meles meles*) to characterize the extent of extra-group paternity, occurring as a consequence of breeding excursions, and to test hypothesized drivers of variation at multiple levels. We jointly estimate parentage and paternity distance (PD; distance between a cub’s natal and its father’s social group), and test whether population density and sex ratio influence mean annual PD. We also model cub-level PD and extra-group paternity (EGP) to test for variation among social groups and parental individuals. Mean PD varied among years but was not explained by population density or sex ratio. However, cub-level analysis shows strong effects of social group, and parental identities, with some parental individuals being consistently more likely to produce cubs with extra-group partners. Group effects were partially explained by local sex ratio. There was also a strong negative correlation between maternal and paternal social group effects on cub paternity distance, indicating source-sink dynamics. Our analyses of paternity distance and EGP indicate variation in extra-group mating at multiple levels—among years, social groups and individuals. The latter in particular is a phenomenon seldom documented and suggests that gene flow among groups may be disproportionately mediated by a nonrandom subset of adults, emphasizing the importance of the individual in driving eco-evolutionary dynamics.

## Teaser

Variation in breeding behavior exists at multiple levels. Based on indirect observations from a genetic pedigree, we found that individual badgers show consistent differences in their tendency to mate with the members of other groups and the distance they travelled to breed. Social groups also differ from each other, with some acting as sinks, both attracting and retaining genes. Average distance travelled also varied annually, but not because of changes in population density or sex ratio.

## INTRODUCTION

Movement of individuals and/or gametes influences the dynamics, persistence, and genetic diversity of spatially structured populations (Ronce 2007). Understanding movement is therefore crucial for wildlife conservation and management as it can determine species distributions (Holt 2003), impact the vulnerability of populations to extinction (Thomas 2000) and play an important role in the transmission of infections (Pope et al. 2007). Behaviors linked to “dispersal,” in the broadest sense of any movement with potential consequences for gene flow (Ronce 2007), are widely viewed as adaptive, allowing individuals to escape from locally intense competition for resources or mates (Daniels and Walters 2000; Matthysen 2005), seek good or compatible genes in potential mating partners (Hamilton 1990; Zeh and Zeh 1996), or avoid inbreeding by leaving the vicinity of related individuals (Greenwood 1980). However, as such movements carry risks as well as benefits, associated behaviors are likely to have evolved under the influence of multiple interacting factors that ultimately shape the balance of costs and benefits (Bowler and Benton 2005; Ronce 2007).

Some of the factors influencing the costs and benefits of movement and dispersal are well documented. For instance, sex (Clarke et al. 1997; Beirinckx et al. 2006; Rabasa and Gutie 2007), age (Dale et al. 2005; Bowler and Benton 2009; Kentie et al. 2014), and density (e.g., Matthysen 2005; Nowicki and Vrabec 2011) are common drivers of variation in many taxa, although density effects can themselves be scale-dependent (e.g., Marjamäki et al. 2013). However, in addition to demographic and ecological effects, it is also becoming apparent that populations can harbor among-individual variation in the tendency to disperse. Our understanding of what drives this variation within animal populations remains limited, although social interactions and behavioral differences (e.g., “personality” variation in exploratory tendency) likely play an important role (e.g., Cote et al. 2010; Patrick et al. 2012; Weiß et al. 2016).

In this study, we employ an indirect approach to test for and investigate sources of variation in breeding excursions in a population of European badgers (*Meles meles*) in southwest England. Temporary excursions relating to mate acquisition are common in many populations but, while they will have important consequences for fine scale gene flow and genetic structure (e.g., among groups), temporary and short-term excursions can be difficult to observe directly. Nonetheless, in the absence of direct observation of movement, indirect inferences on breeding excursions can be made from genetic data. This can be done, for example, by characterizing population genetic structure (or lack thereof; Wilson et al. 2004), or by detecting extrapair or extra-group paternity (hereafter “EGP”), which is commonly seen in birds and mammals (Griffith et al. 2002; Isvaran and Clutton-Brock 2007). Combined with genetic pedigree analysis, the latter approach allows identification of those individuals engaging in, as well as resulting from, extra-group matings, enabling the drivers of among-individual variation to be investigated.

Badgers are a facultatively social species and form social groups at high densities through retention of offspring in natal groups (Kruuk and Parish 1982; da Silva et al. 1994). These social groups, ranging from 1 to 22 individuals of mixed age and sex, form discrete, defended territories containing several communal setts (underground dens). Badgers have a polygynandrous mating system where as many as 7 males and females might breed within a social group annually (Dugdale et al. 2007). While within-population movement is common (e.g., detected in 44% of individuals studied by Rogers et al. 1998), the majority of movements between social groups are temporary, with short-term movements tending to be predominantly between neighboring social groups (Rogers et al. 1998). High rates of EGP (up to 50% reported in high-density populations; Carpenter et al. 2005; Dugdale et al. 2007) are also consistent with an important role for breeding excursions in mediating gene flow, though whether EGP is mediated through transient contact between individuals, or temporary integration of individuals into social groups (or both) is not yet clear.

We use a long-term dataset on individually marked badgers from Woodchester Park (Gloucestershire, England) to reconstruct a genetic pedigree and indirectly estimate breeding excursions. We build on a previous parentage analysis of the population (Carpenter et al. 2005) to reconstruct a pedigree using a larger sample, more markers and more powerful parentage assignment methods. Crucially, for current purposes, we adopt a Bayesian approach to pedigree analysis, which allows us to make better use of spatial and group membership information to improve the number of assigned relationships and our confidence in them (Hadfield et al. 2006). From this we simultaneously estimate both the pedigree structure and the mean distance between the father’s social group and the cub’s natal group (hereafter “paternity distance”) for each annual cohort. We first ask whether paternity distance varies among years as a function of population density and/or sex ratio, before using assigned parent–offspring relationships to test for among-individual (parent) variation in extra-group mating. Finally, noting that from a cub’s perspective, EGP and non-zero paternity distance may reflect temporary excursions by either parent, we ask whether among-parent variation can be explained by known predictors of breeding behavior in other systems, including intrinsic factors (e.g., age, body mass) and social group properties.

## METHODS

### Study population and sampling

The badger population at Woodchester Park (51°42′35′′N 2°16′42′′W), Gloucestershire, UK, has been subject to an ongoing mark-recapture study since 1976. The study area is approximately 11 km^2^ and consists of a steep-sided, wooded valley surrounded by farmland. Here, we utilize data from a 30-year period from 1985 to 2014, for which badgers were trapped and sampled up to 4 times a year. Steel mesh box traps were deployed at active badger setts and set to catch for 2 consecutive nights after a period of 4–8 days of prebaiting with peanuts. Trapped badgers were anesthetized (de Leeuw 2004) prior to examination and at first capture each individual received a unique identifier tattoo on their abdomen. Capture location, sex, age (if birth year known) or age class (adult, yearling, cub, based on size and tooth wear), and body weight were recorded (Delahay et al. 2013). Approximately 20–30 guard hairs were plucked and stored in 80% ethanol for microsatellite genotyping. After a recovery period, all badgers are released at the point of capture. The total trapping dataset is comprised of over 15,000 captures for 3283 individuals. While most badgers are first caught as cubs or yearlings, 19% were first captured as adults and likely represent a minimum estimate of immigration into the population. Social group territorial boundaries were determined for each year of the study by bait-marking (Delahay, Langton, et al. 2000). A total of 45 defined social groups were counted throughout the study period, but from 1996 onwards sampling was focused on 20—25 groups only. Thus, the variation in the number of social groups reflects variation in both sampling effort through time and the configuration of social groups, which occasionally undergo fissions and fusions (though territories are largely stable over time; Delahay, Brown, et al. 2000; Robertson et al. 2014). All work was carried out under license from the UK Home Office and from Natural England.

### DNA extraction and genotyping

Microsatellite data used for parentage analyses have been produced as part of the ongoing Woodchester Park study. For current purposes, we used existing published data (Carpenter et al. 2005) coupled with de novo genotyping at 6 loci described in Carpenter et al. (2003) and Lopez-Giraldez et al. (2007). In brief, individuals trapped between 1986 and 2002 have been genotyped with DNA extraction from hair samples according to protocols outlined in Carpenter et al. (2005), while samples between 2003 and 2014 were genotyped at the NERC Biomolecular Analysis Facility (University of Sheffield, UK) in batches across several time periods using the ammonium acetate extraction method described in Richardson et al. (2001). A minimum of 5 hairs with visible roots were used per individual.

Individuals have been genotyped at between 16 and 22 autosomal microsatellite loci, with slightly different, but overlapping subsets of markers used over the course of the project. We used a 2-μl Qiagen Multiplex PCR reaction (Qiagen Inc., Valencia, USA) and fluorescently-labeled primer sets, before separation of the amplicons on a 48-capillary ABI 3730 DNA Analyzer using Prism set D and a ROX size standard and genotype scoring using GENEMAPPER 3.7. Samples described in Carpenter et al. (2005) were genotyped at 16 loci (Mel 101–117; as described in Carpenter et al. 2003). An additional 6 loci were added to subsequent genotyping efforts (Mel 1, 10, 12, 14, 15, and 116; Carpenter et al. 2003, Lopez-Giraldez et al. 2007) though for 209 individuals born (or captured for the first time) after 2011, markers Mel 15 and 106 were not used. As genotyping has been done in batches over a number of years, samples have been cross-validated by retyping subsets of previously genotyped individuals (min. 15% of samples). This was used to calibrate allele sizes at each locus to ensure consistent scoring across time periods and different sequencers. After scoring genotypes, we tested for deviations from Hardy-Weinberg equilibrium (HWE) and linkage equilibrium (LD) for pairs of loci using 40 unrelated individuals (based on ML-Relate relatedness estimates <0.125) using Genepop 4.4.3 (Raymond and Rousset 1995). P-values for LD tests were corrected to account for multiple tests (false discovery rate; Benjamini and Hochberg 1995). No deviation from HWE (k = 22, alpha = 0.05) or LD (LD: k = 231, alpha = 0.05, adjusted *P* = 0.05–0.0002) were found. Null allele frequencies were estimated using CERVUS 3.0.7 (Marshall et al. 1998) and were <0.1 for all loci. Therefore, all loci were retained. We also estimated mean allelic dropout (e1) and false allele rates (or stochastic sampling error, e2), using a random subset of individuals that were regenotyped and analyzed using PEDANT 1.0 (Johnson and Haydon 2007) (Supplementary Table S1). Overall, genotypes were available for 2204 (out of 2811) trapped individuals, at a mean (±standard deviation [SD]) of 16.1 (±5.1) loci per individual. Across loci the mean observed and expected heterozygosity were 0.56 (SD 0.15) and 0.61 (SD 0.13), respectively, and the mean number of alleles per locus was 4.85 (SD 1.47).

### Parentage analysis

We conducted Bayesian parentage analysis for 1768 genotyped cubs trapped between 1986 and 2014 inclusive, using MasterBayes 2.54 (Hadfield et al. 2006) in R 3.3.0 (R Development Core Team 2016). Relative to most wild birds and mammals in which molecular pedigree reconstruction has been applied, badgers present a particular challenge in that they are largely nocturnal and so difficult to observe. Furthermore, cubs remain underground for the first 12 weeks of life (Roper 2010), and alloparental care may occur at the sett (Dugdale et al. 2010). As such, while maternal identities can often be (reliably) inferred from observation in other species, this is not the case in badgers. In the absence of any known parents, life-history, spatial, and genetic data were used simultaneously to assign paternity and maternity jointly for each cohort of cubs (*n* = 29) and estimate mean annual paternity distance. The final pedigree used in downstream analyses was then compiled based on parental assignments that met a minimum confidence threshold of 80%. For comparison, we also compiled a pedigree structure according to a stricter 95% confidence threshold.

#### Definition of candidate parents and use of spatial data

Parentage assignments were run for each annual cub cohort (*n* = 29). Although neither parent can be determined by observation we follow the approach used in other systems (e.g., Walling et al. 2010; Nielsen et al. 2012) of applying a biologically informed set of criteria to define a nonexcluded list of candidate parents for each cub. For each cohort, candidate mothers were restricted to females aged ≥2 years present in the cub’s natal group (i.e., the group first captured in) in the year of birth, as females are sexually mature as yearlings and, due to delayed implantation (Yamaguchi et al. 2006), can first give birth as 2-year olds. Males were considered candidate fathers (regardless of social group) if they were alive and ≥1 year of age 12 months before the cub was born, to account for delayed implantation. Individuals were designated as belonging to a social group if they were caught within the territory of that group. Individuals recorded in multiple social groups were assigned joint membership to each; in years where individuals were not caught (but were known to be alive from subsequent captures), they were assigned to the social group(s) they were recorded in the preceding year. Only individuals caught as cubs or yearlings (i.e., those with known birth year) were included as offspring in parentage analysis, while badgers first caught as adults are likely to be immigrants and were included only as candidate parents. Since age data were incomplete for badgers that were not caught as cubs or yearlings (distinguishable from adults by size and tooth wear), we assumed adults of unknown age to be 2 years of age at first capture to prevent blanket exclusion from the set of candidate parents (note, this was for parentage assignment only, and assumed ages were not used in subsequent analyses described below). Similarly, where time of death was unknown, individuals were treated as being alive (for purposes of defining status as a potential candidate parent) for 1 year (cubs; Dugdale et al. 2007) or 3 years (adults; Carpenter et al. 2005) after their last capture. Individuals with missing sex or social group data were excluded.

In addition to microsatellite data, our parentage analyses also utilized geographical location data (main sett coordinates for each social group) for all offspring and candidate fathers. Inclusion of nongenetic data is expected to improve assignment where it provides additional information about the likelihood of parentage (Hadfield et al. 2006). For most cohorts (see below), we therefore used (Euclidean) “male distance” between the main sett of the candidate father’s social group and that of the cub’s natal group as a predictor of paternity, which yielded an estimate for each cohort (or year) of the mean paternity distance, i.e., distance between the main sett of the assigned father’s social group and that in which the cub was born. Thus, paternity distance and parentage are jointly estimated from the data in a single analysis (i.e., it is not the case that distance effects on paternity likelihood are first estimated and imposed in a subsequent parentage assignment). Finally, we note that, while more complete genetic sampling of the population should result in greater parentage assignment success (all else being equal), the number of unsampled parents is estimated in a MasterBayes analysis, not specified a priori as an input parameter (as in some likelihood-based methods of parentage assignment). Here we have limited knowledge of the completeness of genetic sampling but certainly trapping does not sample all animals present on any given occasion. Quarterly recapture rates (i.e., across trapping sessions) are known to vary greatly across years, from 0.15 to 0.73 for females and from 0.20 to 0.78 for males (Graham et al. 2013). Approximately 19% of individuals are first trapped as adults, providing an upper bound estimate for the proportion of immigrants to the study area.

#### Parentage assignment settings and diagnostics

Markov chains were run separately for each year (i.e., cub cohort) for 2 million iterations, with a thinning rate of 100 and burn-in period of 500,000. Mismatch tolerance between cub and candidate parent was set to one. Tuning parameters were specified for each cohort to ensure that Metropolis–Hastings acceptance rates were within acceptable limits (0.2–0.5; Hadfield 2012). Per locus genotyping error (e1 and e2; Supplementary Table S1) and allele frequencies calculated based on the full dataset were provided in the model specifications (as direct estimation of error rates by MasterBayes from the data, though possible in principle, is particularly computationally demanding; Hadfield 2012). The presence of unsampled males (per population) and females (per social group) was also allowed for each cohort. Successive samples from the posterior distribution had low autocorrelation (*r* < 0.10) for estimates of unsampled males and paternity distance. Autocorrelation for unsampled females remained high (>0.10) for several cohorts, however, parentage assignments at ≥80% confidence for these cohorts did not differ when a fixed number of unsampled females (one per social group) was used; therefore, all cohorts were retained.

In 6 of the 29 cohorts (1988, 1993, 2001, 2009, 2013, and 2014), inclusion of male distance as a predictor caused problems for the parentage assignment algorithm that we were unable to resolve. The reasons for this remain unknown but could include, for instance, undetected outliers or errors in the spatial data. For these cohorts, parentage assignment was therefore estimated without male distance as a predictor meaning no direct estimate of mean paternity distance was obtained. As including the distance variable is expected to increase confidence in assignments (Hadfield 2012), excluding this variable from pedigree models could affect the resulting parent assignments. In order to account for this, we reran a subset of cohorts (including 339 cubs) without male distance and compared assignments with and without paternity distance estimation. As expected, excluding male distance generally reduced the confidence assigned to a cub’s most likely father, with the result that putative paternities were not assigned in 30 instances, when they had been with models utilising male distance. However, changes in most likely father were only observed for 4 cubs (out of 339). In all 4 cases, most likely candidate fathers failed to meet the 80% confidence threshold for assignment regardless of whether the male distance variable was included. Therefore, based on these comparisons, we expect fewer paternities will have been assigned for the 6 cohorts where the distance variance could not be included, but consider it unlikely that the identity of the most likely father is sensitive to inclusion of male distance in many instances.

### Analysis of breeding excursion proxies

We used the results of our pedigree analysis to extract and model variation in 3 response variables relating to extra-group paternity. First, we modeled among-cohort variation in mean paternity distance as estimated directly by MasterBayes (subsequently denoted PD_*c*_). Second, for each cub with an assigned father, we extracted the individual paternity distance (denoted PD_*i*_), and also defined a binary EGP variable (denoted EGP_*i*_) according to whether the assigned father was from within (0) or outside (1) the cub’s natal group. If a cub was assigned both within- and extra-group paternity by the same father (e.g., where a father was recorded in multiple social groups within a year), the cub was assumed to be within-group offspring. Both PD_*i*_ and EGP_*i*_ are defined for the cub (*i*) and non-zero values therefore reflect movements by the mother and/or the father beyond its own social group. We also note that these individual-level estimates are necessarily derived from an estimated pedigree and thus carry over error associated with parentage assignments to downstream analyses that are not readily accounted for. In this respect, we also note an unavoidable trade-off, regarding analyses of PD_*i*_ and EGP_*i*_, between using assignments made at 80% confidence (increased samples size but higher error rate) or 95% confidence (reduced sample size but lower error rate). Here results from analyses are presented using the lower threshold but parallel analyses based on 95% confidence can be found in Supplementary Materials (Supplementary Tables S6–S8). Overall, qualitative conclusions are consistent between analyses based on the 2 thresholds. Note however that, since MasterBayes estimates a full posterior distribution for PD_*c*_, uncertainty in this parameter could be readily accounted for in our analysis of among-cohort variation.

#### Among-cohort variation in annual mean paternity distance

**Figure 1:**
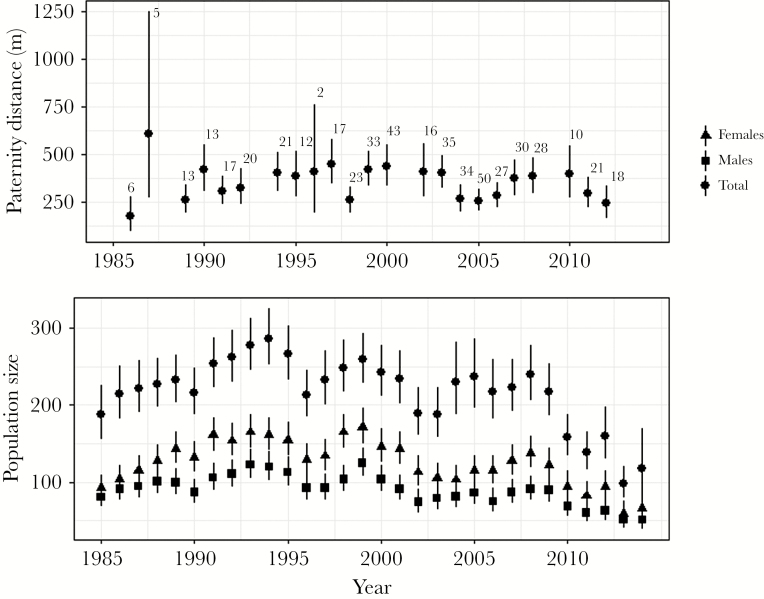
Top: Annual modal paternity distance (PD_*c*_) estimated for each of 23 cohorts by MasterBayes (Hadfield et al. 2006) during pedigree reconstruction. Lines represent 95% credible intervals. Numbers above points represent the number of cubs assigned parentage in each year. Bottom: Total population size and number of males and females estimated in program MARK for each year of the study, based on 20 core social groups with consistent capture records. Bars represent standard errors.

Our MasterBayes analyses generated estimated posterior distributions (15,000 values per cohort) of PD_*c*_ for 23 cohorts caught between 1986 and 2014 (Figure 1). As noted above, in 6 years (1988, 1993, 2001, 2009, 2013, 2014), inclusion of spatial data in the pedigree assignment step proved problematic, so no estimates of PD_*c*_ are available. Using a simple multiple regression model of PD_*c*_ we tested whether total population size or population sex ratio, determined by dividing the number of males by total population size (as defined below), explained variation in mean paternity distance. We also included a (linear) effect of year to test for any systematic trend in PD_*c*_ across the study timeline. All 3 variables were mean centered to ease interpretation of the intercept (i.e., as predicted PD_c_ at mean population size, sex ratio, and year). Because sampling effort for some social groups varied across years, proxies of total population size and population sex ratio values for each year were estimated using the POPAN model in the program MARK 8.2 (White and Burnham 1999) using capture data from 20 “core” social groups with consistent trapping efforts across all years. Graphical representation of annual mean estimates for population size and numbers of males and females can be found in Figure 1b. Badgers with missing sex information (*n* = 2) were excluded from this analysis. In order to integrate across uncertainty in annual mean paternity distance estimation, our regression model was applied to the full posterior distributions of PD_*c*_ for each cohort, allowing estimation of 95% credible intervals (CI) for the partial regression coefficients. These were considered significant if 95% CI did not span zero.

#### Among-individual and among-group variation in paternity distance and extra-group paternity

Using the program ASReml 3.0 (VSN International Ltd., Hemel Hempstead, UK), we fitted mixed effects models of PD_*i*_ (i.e., Euclidean paternity distance measured in meters), and EGP_*i*_, a binary variable assigning the offspring of each male as either within (0) or extra (1) group. For both response variables, a Gaussian error structure was assumed but PD_*i*_ was natural log-transformed prior to analysis to reduce positive skew in residuals. While noting that the Gaussian assumption cannot be strictly true for bounded (ln PD_*i*_) or binary (EGP_*i*_) response variables, inspection of model residuals showed it to be a reasonable approximation here (Supplementary Figure S2). We therefore chose this approach as being more pragmatic than, for instance, Bayesian implementation of generalized mixed models as it more readily allows inference on, and modeling of hypothesized covariance between, random effects (see below). Both variables were then scaled to standard deviation units (SDU) to ease interpretation of results.

For both response variables, models included fixed explanatory variables of maternal age, maternal body mass, maternal group size, and maternal social group sex ratio (as linear effects) and the corresponding paternal variables. Social group sizes (mean 6.4 SD ±3.6) reflect numbers of resident yearlings and adults (i.e., reproductively active individuals) in the cub’s conception year, where group residency is determined from capture records each year following Vicente et al. (2007). Social group sex ratios are calculated as the number of males divided by the total number of adult group members, representing the proportion of males in each group (mean 0.4 SD ±0.2). These measures exclude cubs and transient nonresidents (based on criteria used by Vicente et al. 2007) caught within social group boundaries, but represent a baseline measure for the density of potential breeders encountered by individuals in their social group. Body mass was included to test for size-dependence of extra-group paternity and for individuals with more than one weight measurement within a year, the mean of these was used. Note that we also fitted the models using a standardized measure of body condition, the scaled mass index (SMI; Peig and Green 2009), in place of body mass. In principle, this might better account for sexual dimorphism and seasonal variation in body mass (Peig and Green 2010; Beirne et al. 2015). However, in practice, qualitative conclusions of the analyses were unaltered, and since use of SMI in place of body mass resulted in a 16% reduction in sample size, only the results of analyses using body mass are presented here (results for SMI analysis can be found in Supplementary Tables S3–S5). Significance of fixed effects was determined using conditional Wald F-tests implemented in ASReml (with denominator degrees of freedom calculated following Kenward and Roger 1997).

Year (as a factor), maternal and paternal identities, and maternal and paternal social group IDs were included as random effects in the models. This allowed us to partition variance in PD_*i*_ and EGP_*i*_ to assess the relative importance of individual and group-level effects (conditional on fixed effects). We make the standard assumptions that random effects are normally distributed with means of zero and variances to be estimated. For ease of interpretation, variance components were standardized to intraclass correlations (ICC) by dividing by phenotypic variance (determined as the sum of all variance components). ICC are thus interpretable as individual and group repeatabilities (R) for random effects relating to parental individuals and their social groups (Nakagawa and Schielzeth 2010). In addition, we explicitly modeled a covariance term between the maternal and paternal social group identity effects. The strength and sign of this relationship is biologically informative since, for instance, if groups vary in EGP in a nonsex-specific way we predict a positive covariance. Conversely, since cub natal and maternal social groups are the same, if EGP follows a source-sink dynamic with respect to genetic consequences (i.e., some groups are net importers of genes and some net exporters) we predict a negative relationship.

Statistical inference on random effects was by likelihood ratio test comparison of the full model to reduced formulations in which (co)variance components arising from the tested random effects were assumed absent. Twice the difference in log-likelihood between full and reduced models was assumed to have a χ^2^- distribution, and we conservatively (see Visscher 2006) assume the degrees of freedom (df) equal to the number of additional parameters in the full model.

The analyses described above were conducted using all available PD_*i*_ and EGP_*i*_ observations based on the 80% confidence threshold for parentage assignment. To assess sensitivity of results to this choice of confidence threshold, we repeated the analyses using only parentage assigned at 95% confidence. While the higher threshold should reduce “measurement error” in PD_*i*_ and EGP_*i*_ arising from erroneous assignments, it also reduced sample size for analyses of these variables. Overall, conclusions regarding individual and group-level variation remained broadly the same. Some inflation of variance components occurred in models using the higher threshold, and there were also some changes to the significance of fixed effects. Full results of these additional analyses are reported in the electronic supplement (Supplementary Tables S6–S8) and commented on, where appropriate, below.

## RESULTS

### Parentage analysis

In total, pedigree reconstruction resulted in 617 cubs being assigned at least one parent (35% of genotyped cubs included in the analyses), representing 29 cohorts and 6 generations (see Supplementary Figure S1 for visual representation). Out of these, 556 (89%) cubs were assigned both parents, while 23 (4%) were assigned only a mother and 40 (7%) only a father. Overall, the 1175 parental relationships (579 maternities and 596 paternities) were represented by 239 fathers and 278 mothers. Among these, half-sibship sizes (mean ± SD) varied from 1 to 11 (2.08 ± 1.53) for mothers and 1–14 (2.49 ± 2.37) for fathers, with a total of 638 maternal and 1113 paternal sibships out of which 186 were full sibships. Additionally, 189 and 191 maternal grandmaternal and -paternal, as well as 155 and 161 paternal grandmaternal and -paternal links were present. Based on successful maternal assignments, mean litter size was 1.24 (range 1–3), which is slightly lower than previous reports for this and other populations (1.4–1.5; Carpenter et al. 2005; Dugdale et al. 2007; Annavi et al. 2014). Out of 101 litters of more than one cub, 23% (compared to a previous estimate of 16%; Carpenter et al. 2005) were multiple paternity litters, comprising 18 litters of *n* = 2 and 4 of *n* = 3 contributed to by 2 different fathers, and one of *n* = 3 with each cub assigned a different father. Parent–offspring assignments covered 37 social groups out of the 45 represented in the full database. Based on the parent–offspring assignments made, the mean rate of extra-group paternity over the 29 years was 37.1% (SD ±18.4). The relatively small proportion of assignments likely reflects the lack of strong prior information on maternity in badgers. Certainly, this greatly reduces power, and so the number of assignments, relative to paternity assignment when the mother is already known (Jones et al. 2010). Incomplete sampling of candidate parents is likely to be another contributing factor. The number of unsampled candidate parents estimated by MasterBayes varies considerably between cohorts with a median (range) of 0.819 (0.359–0.628) females per group, and 20.4 (5.13–239) males in the whole study area (Supplementary Table S9). Out of the total parent–offspring assignments accepted at ≥80% confidence, 34% and 19% were assigned with ≥90% and ≥95% confidence, respectively.

### Among-cohort variation in mean annual paternity distance

Across the 23 cohorts for which spatial data could be included in the parentage assignment, point estimates of PD_c_ obtained as the mean of the posterior distributions for each cohort varied from 173 m (95% CI, 93–275 m) to 608 m (95% CI, 270–1249 m) with a mean of 354 m (SE ±19.6) across cohorts. Despite relatively high uncertainty around some annual estimates, nonoverlapping credible intervals for some pairwise comparisons indicate significant annual variation in PD_*c*_ (Figure 1a). However, this variation was not related to any of the explanatory variables (population size, sex ratio, or year treated as a continuous variable to characterize any trend) tested in our multiple regression model (Table 1).

**Table 1:** Estimated effects of population size, sex ratio, and cohort (year) on modal annual paternity distance (PD_c_)

	**Estimate**	**95% credible interval**
**Intercept**	332.43	319.90−382.60
**Population size** ^†^	0.36	−0.67–1.15
**Sex ratio** ^‡^	−331.43	−1706.30–1743.66
**Year**	0.44	−7.81–4.74

Estimates are from multiple regression with uncertainty integrated over the full posteriors of annual PD_c_ (see main text). Predictors were mean centered for analysis.

^†^Annual estimate of the number of badgers in Woodchester Park, based on 20 “core” social groups with consistent capture records.

^‡^Calculated from annual population size estimates as the number of males divided by total population.

### Among-individual and among-group variation in paternity distance

**Figure 2:**
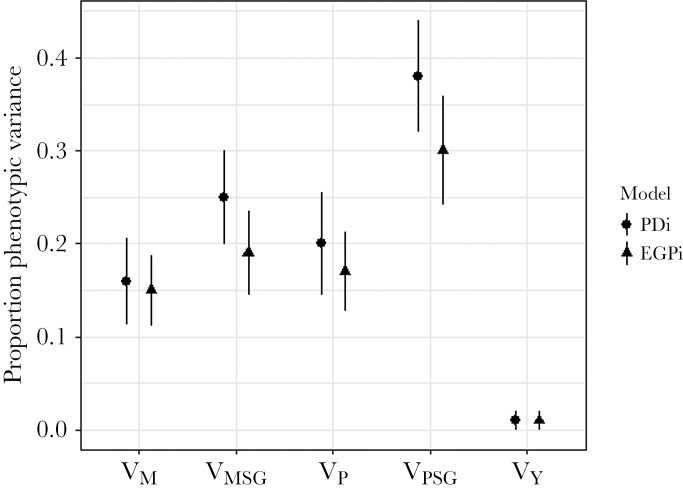
Estimated intraclass correlations (i.e., proportion of total phenotypic variance calculated by dividing each component by the sum of all variance components) for each random effect in models of PD_i_ and EGP_i_. Bars represent standard errors. M and P denote maternal and paternal individuals, while MSG and PSG denote the corresponding maternal and paternal social groups.

Our mixed model analysis of PD_*i*_ indicated no significant effects of parental age, weight, or group size (neither maternal nor paternal variables; Table 2). Maternal social group sex ratio, on the other hand, had a significant negative effect on paternity distance (Table 2), indicating that cubs from maternal social groups (i.e., cub’s natal group) with a higher proportion of males have lower paternity distances on average. Paternal social group sex ratio showed the opposite trend, but the effect was not significant (*P* > 0.05). Testing the random effects provided evidence of significant among-individual variation in PD_*i*_ for both mothers (among-mother repeatability, denoted *R*_M_ = 0.16 SE ±0.05, χ^2^ = 40.29, *P* < 0.001) and fathers (among-father repeatability, denoted *R*_P_ = 0.2 SE ±0.06, χ^2^ = 35.82, *P* < 0.001) (see Figure 2). Comparison of the full model fit to one in which maternal and paternal identity variance components were constrained to be equal provided no significant evidence against the null hypothesis that mother and father explain equal variance in cub PD_*i*_ (χ^2^ = 0.38, *P* = 0.5). The random effect of year was estimated at c. 1% of the variance and was not significant.

**Table 2:** Estimated fixed effect coefficients (standard error) and Wald *F*-tests from mixed models of log-transformed PD_i_ and EGP_i_ (see main text for details)

	**Log(PD**_**i**_)				**EGP**_**i**_			
	**Estimate (SE)**	**df**	***F***	***P***	**Estimate (SE)**	**df**	***F***	***P***
Intercept	−0.72 (0.15)	1, 214.7	24.56	**<0.001**	0.72 (0.14)	1, 226.9	74.97	**<0.001**
Age_M_	−0.45 (0.15)	1, 533.1	0.09	0.76	−0.52 (0.15)	1, 534.0	0.12	0.73
Body mass_M_^†^	−0.61 (0.13)	1, 302.8	0.22	0.63	−0.66 (0.13)	1, 304.1	0.26	0.61
Group_size_MSG_	0.94 (0.18)	1, 456.9	0.28	0.59	0.96 (0.18)	1, 443.0	0.29	0.59
Sex_ratio_MSG_^‡^	−0.74 (0.22)	1, 531.5	10.97	**<0.001**	−0.82 (0.22)	1, 524.2	13.55	**<0.001**
Age_P_	0.28 (0.2)	1, 516.7	2.11	0.15	0.30 (0.2)	1, 517.3	2.4	0.12
Body mass_P_^†^	−0.59 (0.12)	1, 213.4	0.25	0.62	−0.56 (0.19)	1, 215.0	0.23	0.64
Group.Size_PSG_	−0.12 (0.18)	1, 537.4	0.44	0.50	−0.12 (0.18)	1, 531.9	0.43	0.51
Sex_ratio_PSG_^‡^	0.43 (0.24)	1, 538.1	3.21	0.08	0.50 (0.24)	1, 536.0	4.48	**0.04**

Response variables were standardized into standard deviation units (SDU) prior to analysis. M and P denote maternal and paternal individuals, while MSG and PSG denote the corresponding maternal and paternal social groups. df stands for degrees of freedom. *P*-values in bold denote significance at alpha 0.05.

Full models fitted for each response were y ~ μ + Age_M_ + Body_Mass_M_ + Group_size_MSG_ + Sex_ratio_MSG_ + Age_P_ + Body_Mass_P_ + Group_size_PSG_ + Sex_ratio_PSG_ + *M* + *P* + *MSG* + *PSG* + *Year* where italic font denotes random effects and y is either log(PD_i_) or EGP_i._

^†^Mean body mass for parental individuals with multiple weight measurements within year of cub’s birth.

^‡^Calculated as number of males divided by group size where group size is males plus females.

**Figure 3:**
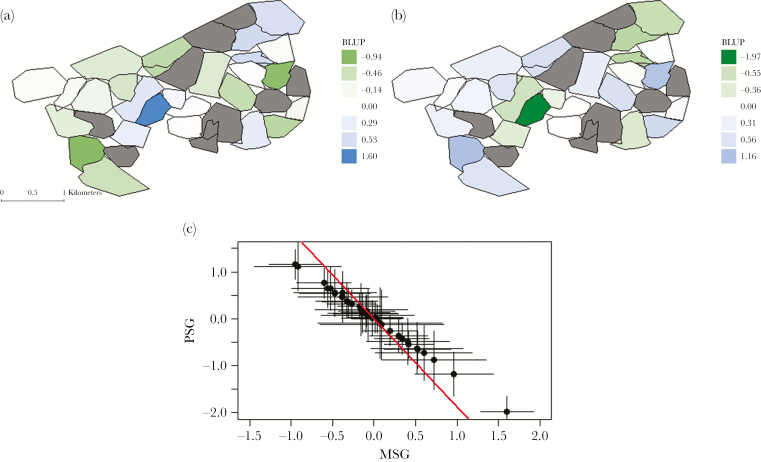
Spatial representation of (a) maternal and (b) paternal social group effects and (c) the relationship between them. Effects are predicted from the mixed model of log-transformed PD_i_ (see main text) using best linear unbiased prediction (BLUP) while the spatial configuration of social group territories illustrated is derived from a bait-marking survey in 1993 (when the maximum number of social groups were present). Six social groups included in current analyses are not shown on panels (a) or (b) due to missing bait-marking data, while grey shaded territories correspond to groups with no parentage assigned. Error bars in panel (c) denote ± standard error and the regression line (red) slope is calculated directly from the model (co)variance estimates as COV_MSG.PSG_/V_MSG._ MSG and PSG denote maternal and paternal social groups.

Parental social group identities also explained significant variation in PD_*i*_, with group-level repeatabilities of *R*_MSG_ = 0.25 (SE ±0.05; χ^2^ = 58.2, *P* < 0.001) and *R*_PSG_ = 0.38 (SE ±0.06; χ^2^ = 64.5, *P* < 0.001), where MSG refers to maternal, and PSG to paternal social group (Figure 2). The difference in the proportion of variance in PD_*i*_ explained by PSG compared to that of MSG was marginally nonsignificant (χ^2^ = 3.43, *P* = 0.06). There was a strong negative covariance between maternal and paternal group identity effects, which corresponds to a correlation (±SE) of *r*_MSG.PSG_ = −0.99 (±0.03; χ^2^ = 39.3, *P* < 0.001; Figure 3c). Thus, social groups in which resident females (males) are more likely to mate with males (females) from further away are the same groups in which resident males (females) are less likely to mate with females (males) from further away. To visualize this pattern better, and the among-group variation in PD_*i*_ generally, we extracted the group-level random effect predictions (best linear unbiased predictors or [BLUPs], see Supplementary Table S2), which represent the predicted deviation of each (maternal and paternal) social group from the mean paternity distance, and overlaid them on a spatial map of the study area (Figure 3). This confirms that PSG with longer-than-average paternity distances, correspond to MSG with shorter-than-average paternity distances. Biologically, this is consistent with source-sink dynamics where some groups both retain resident male genes as well as attracting extra-group paternity, however, under the current methodology it is not possible to discern whether it is primarily driven by physical movement of males, females, or both. Note that while the sources of among-group variation are unknown, we highlight that estimates here are conditioned on group size and sex ratio, the latter having some effects as described above.

### Among-individual and among-group variation in extra-group paternity

Analysis of EGP_*i*_ yielded broadly similar insights to our model of PD_*i*_, although paternal, as well as maternal, social group sex ratio had significant effects on extra-group paternity (Table 2). Similar to PD_*i*_, the effect was negative for maternal, and positive for paternal group sex ratio. Thus, there is lower extra-group paternity among offspring in groups with higher male to female ratios. Other fixed effects were nonsignificant (Table 2). Maternal and paternal ID had significant repeatabilities (*R*_M_ = 0.15 ± 0.04, χ^2^ = 40.61, *P* < 0.001; *R*_P_ = 0.17 ± 0.04, χ^2^ = 35.34, *P* < 0.001) indicating consistent differences among individuals of both sexes in their tendency to have offspring with extra-group partners (Figure 2). Social group-level effects were also significant and again almost perfectly negatively correlated (*r*_MSG.PSG_ = −0.99 SE ±0.03; Table 3, Figure 3). Differences in the amount of variance explained by maternal versus paternal identity, and MSG versus PSG were not significant, while year explained only a small (and nonsignificant) amount of variance in EGP_*i*_ (Table 3).

**Table 3:** Estimated (co)variance components (standard error) associated with random effects in mixed models of EGP_*i*_ and log-transformed PD_*i*_

		**log(PD**_***i***_)				**EGP**_***i***_		
	**Variance (SE)**	**df**	χ^**2**^_**1**_	***P***	**Variance (SE)**	χ^**2**^_**1**_	**df**	***P***
**V**_**year**_	0.02 (0.02)	1	3.22	0.07	0.02 (0.03)	2.83	1	0.09
**V**_**M**_^†^	0.26 (0.05)	1	40.29	**<0.001**	0.26 (0.06)	40.61	1	**<0.001**
**V**_**P**_^†^	0.31 (0.06)	1	35.82	**<0.001**	0.31 (0.06)	35.34	1	**<0.001**
**V**_**MSG**_^‡^	0.39 (0.15)	2	58.16	**<0.001**	0.34 (0.13)	55.00	2	**<0.001**
**V**_**PSG**_^‡^	0.59 (0.21)	2	64.54	**<0.001**	0.54 (0.19)	62.91	2	**<0.001**
***COV***_***_MSG,PSG_***_	*−0.48 (0.17)*	*1*	*39.33*	***<0.001***	*−0.43 (0.15)*	*36.84*	*1*	***<0.001***
**V**_**R**_	0.32 (0.04)	-	-	-	0.32 (0.04)	-	-	-

Statistical inference of random effects is by likelihood ratio test results (see main text for details). M and P denote maternal and paternal individuals, while MSG and PSG denote the corresponding maternal and paternal social groups. *P*-values in bold denote significance at alpha 0.05.

^†^Not significantly different from each other (logLRT, PD_i_: χ^2^ = 0.38, *P* = 0.5 EGP_i_: χ^2^ = 0.28, *P* = 0.6).

^‡^Not significantly different from each other (logLRT, PD_i_: χ^2^ = 3.43, *P* = 0.06, EGP_i_: χ^2^ = 3.68, *P* = 0.06).

## DISCUSSION

We examined variation in breeding excursions using pedigree-derived information on extra-group paternity and paternity distance in a wild population of badgers. We found evidence that cohort mean paternity distance (PD_c_, the mean distance between the social groups of fathers and their cubs) varied among years. Contrary to our predictions, this among-cohort variation in PD_*c*_ was not explained by annual variation in population size or sex ratio, nor did we see any systematic temporal trend in paternity distance over the study period. However, individual (cub) level analyses showed significant among-parent (both mother and father) and among-social group variance in breeding excursions, with the latter contributed to (but not fully explained) by differences in group sex ratios. Below we discuss these findings in the context of the wider literature, focusing on their implications for ecological and evolutionary dynamics.

### Among-cohort variation in average paternity distance

Our point estimates of PD_*c*_ varied considerably among years, suggesting temporal variation in the tendency of badgers to undertake breeding excursions. However, there was no systematic trend over time and cohort variation was not explained by changes in the size or sex ratio of the Woodchester Park population as a whole. A post hoc analysis of PD_i_ and EGP_i_ with population-level estimates included as additional predictors also revealed no significant effects of population size or sex ratio. Year-to-year variation in PD_*c*_ therefore remains unexplained at present, but could plausibly be linked to other variables such as weather conditions, relatedness and neighboring group composition, all of which are known to influence movement, activity and dispersal in badgers (Annavi et al. 2014; Noonan et al. 2014), but which were not investigated here. More generally, the absence of population size effects on PD_*c*_ contrasts somewhat with previous studies. In badgers and other species (e.g. Møller 1991; Mougeot 2004; Annavi et al. 2014), local density-dependence has been reported in rates of extra-group paternity—a pattern often linked to changes in mate guarding behavior (e.g., Møller 1991; Kokko and Rankin 2006; Isvaran and Clutton-Brock 2007), though evidence for mate guarding in badgers is limited (Dugdale et al. 2007). Variation in movement distance has also been linked to population density in badgers (Frantz et al. 2010; Byrne et al. 2014), and is sensitive to local density reductions from culling (Tuyttens, Delahay, et al. 2000; Tuyttens, Macdonald, et al. 2000; Pope et al. 2007). However, we note that paternity distance is considered a proxy for movements relating specifically to breeding excursions here. Certainly, the processes governing rates of breeding excursions may differ from those influencing other types of movement making direct comparisons difficult.

There are also several other explanations for the apparent discrepancy between our results and these previous findings. First, it is possible that among-year density variation in the current study is not sufficient to reveal a density-dependent response, as Woodchester Park has one of the highest recorded densities (25 adults/km^2^) of badgers throughout the species’ range (Rogers et al. 1997) and the habitat may be saturated. However, population fluctuation over the period of this study suggests this is not the case, as population size increased in some years. Second, it is possible that the (overall) population density measure used here doesn’t capture variation at the correct scale to reveal density-dependence. The latter appears to be the case for sex ratio, with temporal variation in population-level PD_*c*_ not being predicted by population sex ratio, but local (i.e., group) sex ratios contributing to spatial variation in EGP_*i*_ and PD_*i*_ defined at individual (cub) level (discussed further below). However, parallel local density effects (modeled as social group size effects) did not contribute to spatial variation in either EGP_*i*_ or PD_*i*_. An additional consideration is the fact that the lack of a clear density-dependent pattern could conceivably be an artifact of the study scale, as high-density populations (such as Woodchester Park) typically involve sampling over smaller spatial areas and may therefore miss longer distance movement (Byrne et al. 2014). Finally, we note that the large proportion of unresolved parentage across the study period, as indicated by the relatively low number of parentage assignments (35% cubs assigned parent(s)), may well have resulted in a lack of power to distinguish density and sex ratio effects on cohort mean paternity distance.

### Among-group variation in cub PD_i_ and EGP_i_

Analysis of cub-level proxies of (parental) breeding excursions revealed several important sources of variation. Parental social group sex ratios influenced both EGP_*i*_ and PD_*i*_. Although we note that the effect of PSG sex ratio on PD_*i*_ was not statistically significant in the main analysis presented, it was significant when we refitted our model using only those paternity distances inferred from assignments at the 95% confidence threshold (see Table S6). Cubs had higher PD_*i*_ (on average) and were more likely to have an extra-group father if born into less male-biased social groups. Conversely, cubs born in groups with more male-biased sex ratios were more likely to be fathered by within-group males. These results are consistent with earlier analysis of trapping data in Woodchester Park in which Rogers et al. (1998) concluded that males preferentially move to groups with a higher proportion of females. Woodroffe et al. (1993) also found that the peak of these temporary excursions coincides, for both males and females, with female estrus while in the Wytham Wood (Oxfordshire, UK) badger population, while, similar to Woodchester Park, higher numbers of within-group males were associated with lower rates of EGP (Annavi et al. 2014). Taken together, these results are consistent with ongoing mate guarding by males (antikleptogamy hypothesis; Robertson et al. 2014) although they do not provide direct evidence. Although previous studies have thus emphasized the role of males in breeding excursions, we stress that our indirect inferences from paternity distance and extra-group paternity do not allow us to discriminate between male and female movements. Temporary excursions by both sexes are possible and our results could reflect important variation in female mating behavior in response to mate availability. For instance, females may be less inclined to seek extra-group matings in male-biased groups if they have greater choice of partners. Nevertheless, the relative importance of contributing factors (e.g., avoidance of male–male competition, female choice for extra-group males, inbreeding avoidance by either sex) is not clear (although see Annavi et al. 2014).

After accounting for sex ratio (and group size) effects, parental social group identities together account for more of the remaining variance in cub PD_*i*_ and EGP_*i*_ (63% and 49%, respectively) than any other variance component. Further, the strong negative correlation between maternal and paternal group identity effects in both models indicates that maternal groups that predispose to high paternity distance are the same as the paternal groups predisposed to low paternity distance. These social group identity effects are not readily explained as a simple consequence of, for example, (relative) distances between groups or edge effects. In the former case, a positive correlation between maternal and paternal social groups would be present, while, in the latter, groups at the edges of the study area would be expected to have below average PD_*i*_. This is because we expect failure to assign paternity to cubs sired by unsampled males from outside the study area, such that edge effects are likely to cause downward bias in average PD_*i*_ and EGP_*i*_ for peripheral maternal groups. However, no such pattern is readily apparent in our analysis (see spatial maps of group effects on cub paternity distance in Figure 3).

Thus, while reiterating the earlier caveat that some long-distance movements may be missed by our analysis, among-group variation in cub paternity distance is not readily explained as an artefact here. Rather the emerging picture is one of source-sink dynamics, where some social groups are more “attractive” than others thus both retaining and drawing in male genes. From the male’s point of view, this could signal variation in some unknown aspect of “quality” among females from different social groups, which itself may be mediated by spatial variation in resource availability (e.g., food, setts) that determine habitat preferences of females. Conversely, the observed pattern could reflect variation in female mating preferences if “attractive” males are spatially clustered. Spatial variation in habitat quality has previously been linked to differences in group size across Woodchester Park (Delahay et al. 2006) and is certainly a plausible hypothesis for explaining among-group differences “attractiveness,” although variance explained by parental social group identities is estimated here conditional on a set of fixed effects including group size. Furthermore, group size itself was not a significant predictor of either response variable in the main analyses presented based on parentage assignments made at 80% confidence. However, using the more stringent assignments threshold of 95%, group sizes did have a significant effect. Given statistical support for group size effects is thus rather equivocal we draw no strong conclusions about its role. However, at least in a qualitative sense it is worth pointing out that PD_*i*_ and EGP_*i*_ seem to increase with paternal group size and decrease with maternal group size.

Similar variation has been recorded in great cormorants (*Phalacrocorax carbo sinensis*), where Minias et al. (2016) found higher rates of extrapair paternity in the periphery than in the centre of a nesting colony. This pattern was not explained by density but by variation in mate quality, as indicated by nest site location. Habitat structure has also been shown to influence rates of extrapair paternity, for instance, in blue-footed boobies (*Sula nebouxii*), by restricting movements within the colony (Ramos et al. 2014). Although our results, as well as results from previous studies (Rogers et al. 1998; Carpenter et al. 2005), suggest that movement in this population is focused around neighboring social groups, with an average PD_C_ of 358 m and a nearest neighbor distance between social group main setts of 355 m (SD 84) m, habitat structure per se is unlikely to influence movement in this population, spatial structuring (particularly of females) instead being mediated by resource availability (da Silva et al. 1994; Delahay et al. 2006).

### Among-individual variation in cub PD_i_ and EGP_i_

In addition to social group effects, we found that there was repeatable variation among both mothers and fathers for cub PD_*i*_ and EGP_*i*_. The most parsimonious interpretation of these results is that there is among-individual variation, in both sexes, for breeding behavior. This interpretation is in line with trapping-based inferences for the Woodchester Park badger population (Rogers et al. 1998), as well as studies of other taxa. For instance, Whittingham et al. (2006) found the proportion of extrapair young produced to be highly repeatable for female tree swallows (*Tachycineta bicolor;* intraclass correlation, *r* = 0.83). In coal tits (*Parus ater*), the proportion of extrapair young showed repeatability in both sexes among the same social pairing (*r* = 0.33 and 0.47 for males and females, respectively; Dietrich et al. 2004). Conversely, breeding excursions were found not to be a repeatable behavior in female roe deer (*Capreolus capreolus*; Debeffe et al. 2014). Among-individual differences in other dispersal and exploratory behaviors have also been recorded for spiders (Bonte et al. 2009; Johnson et al. 2015), fish (Harrison et al. 2015), amphibians (Cosentino and Droney 2016), and birds (Reid et al. 2011a; Patrick et al. 2012; Grist et al. 2014). Thus, among-individual variance in PD_*i*_ and EGP_*i*_ could be linked to both reproductive decision making (i.e., individuals varying in their propensity/ability to seek or obtain extra-group matings), and more general exploratory traits influencing encounter rates between badgers from different groups. Regardless, a further aspect of our analysis worth noting is that similar levels of variation in cub PD_*i*_ and EGP_*i*_ were explained by maternal and paternal identities. Thus, whether gene flow from breeding excursions is being mediated primarily by variation in movement per se, or by reproductive decision making, both sexes appear to have an equal impact.

Our analyses have not clearly identified the underlying source(s) of among-individual variance in (parental) mating behavior. Neither size nor age (of either parent) significantly predict PD_*i*_ and EGP_*i*_ in the main analyses, although we note that using the 95% confidence pedigree the positive effects of paternal age on both response variables are statistically significant (Supplementary Table S4). This suggests that older males tend to produce more extra-group offspring and make longer breeding excursions (or mate with females that do), though this conclusion remains tentative. In a broader sense, among-individual variation will reflect the fact that individuals experience different environmental conditions (e.g., maternal effects, food availability, social status) even within groups and years (which were both modeled separately), although genetic variation may also be present. Dispersal distance has been shown to be heritable in a free-living population of great tits (*Parus major*; *h*^2^ = 0.15 SE ± 0.006; Korsten et al. 2013), as has EGP rate in in female, but not male, song sparrows (*Melospiza melodia*; Reid et al. 2011a, 2011b). It is, therefore, possible that the among-individual variance found here has a partial genetic basis. In fact, the pedigree will facilitate testing this, although it would best be achieved through quantitative genetic modeling of independently obtained trapping data.

## CONCLUSIONS

We have used a genetic pedigree to characterize variation in paternity distance and extra-group paternity in a high-density badger population. We show there to be variation among years and social groups, but also among-parental individuals (both mothers and fathers) within groups. Although effects of social group sex ratio (and potentially group size and paternal age) were detected, in general this variation is not readily explained by life-history and social correlates. Among-group variation appears to follow a pattern of source-sink dynamics, suggesting that some social groups are more attractive to extra-group partners than others, though levels of among-parental variation in our metrics were similar across the sexes. Not readily explained by age or body size, it is possible that genes as well as individual-specific (rather than group level) environmental factors contribute to among-individual variation although this remains to be tested. Individual-level differences can have important consequences for many ecological and evolutionary processes, and our results highlight the fact that individuals can vary consistently in their mating behavior. Together, these results emphasize the importance of including individual-level variation in evolutionary models of animal movement and mating behavior, as well as management and conservation measures.

## FUNDING

This work was supported by a Natural Environment Research Council. P.H.M. was funded by a NERC industrial Case Studentship awarded to A.J.W., R.D., and R.A.M. (grant numbers NE/L009897/1, NE/M004546/1).

## AUTHOR CONTRIBUTIONS

A.J.W., R.A.M., R.D., and P.H.M. designed research; P.H.M performed research; T.B. provided facilities and support for the molecular work; D.A.D. assisted with genotyping and data validation; R.D. contributed data; P.H.M. analyzed data with A.J.W. and H.L.D; P.H.M. lead the write-up with input from all authors.

## Supplementary Material

Supplementary Data
