# Supplementary material for: Individual variation and the source-sink group dynamics of extra-group paternity in a social mammal
Source: Behav Ecol. 2019 Jan 14;30(2):301–12. doi: 10.1093/beheco/ary164 (PMC6450204; doi:10.1093/beheco/ary164)

**This file contains supplementary tables for the article:**

**Individual variation and the source-sink group dynamics of extra-group paternity in a social mammal**

Paula H. Marjamäki*^1,2^, Hannah L. Dugdale^3^, Deborah A. Dawson^2^, Robbie A. McDonald^4^, Richard Delahay^5^, Terry Burke^2^**,** Alastair J. Wilson^1^

^1^Centre for Ecology and Conservation, University of Exeter, Penryn Campus, Penryn, Cornwall, TR10 9FE, UK

^2^NERC Biomolecular Analysis Facility, Department of Animal and Plant Sciences, University of Sheffield, Western Bank, Sheffield, S10 2TN, UK

^3^School of Biology, Faculty of Biological Sciences, University of Leeds, Leeds, LS2 9JT, UK

^4^ Environment and Sustainability Institute, University of Exeter, Penryn Campus, Penryn, Cornwall, TR10 9FE, UK

^5^National Wildlife Management Centre, Animal and Plant Health Agency, Woodchester Park, Gloucestershire, GL10 3UJ, UK

*Corresponding author:

Paula H. Marjamäki

Centre for Ecology and Conservation, University of Exeter, Penryn Campus, Penryn, Cornwall, TR10 9FE, UK

Tel: 07527913031

Email: [pm343@exeter.ac.uk](mailto:pm343@exeter.ac.uk)

Table S1. Per locus mean allelic dropout (e1) and false allele or stochastic sampling error rates (e2), estimated using PEDANT 1.0 (Johnson & Haydon 2007) using 209 individuals for which repeat genotypes were available. Loci for which estimated error was zero, and those for which estimation was not possible (Mel15 & 106) due to lack of repeat genotypes, the default rate of 0.005 was used (Hadfield 2014).

| **Locus** | **E1** | **95% CI** | **E2** | **95% CI2** |
| --- | --- | --- | --- | --- |
| Mel1 | 0.03 | 0.005-0.08 | 0.006 | 0.0006-0.02 |
| Mel10 | 0.03 | 0.005-0.1 | 0 | 0-0.02 |
| Mel12 | 0.1 | 0.07-0.2 | 0.07 | 0.05-0.1 |
| Mel14 | 0.02 | 0.006-0.04 | 0.03 | 0.01-0.04 |
| Mel15 | 0.005 | - | 0.005 | - |
| Mel101 | 0.1 | 0.03-0.2 | 0.02 | 0.002-0.06 |
| Mel102 | 0.02 | 0.006-0.05 | 0 | 0-0.009 |
| Mel103 | 0.02 | 0.0009-0.07 | 0.03 | 0.006-0.06 |
| Mel104 | 0.03 | 0.008-0.08 | 0.01 | 0.001-0.04 |
| Mel105 | 0.03 | 0.01-0.05 | 0.05 | 0.03-0.07 |
| Mel106 | 0.005 | - | 0.005 | - |
| Mel107 | 0.01 | 0.002-0.05 | 0 | 0-0.007 |
| Mel108 | 0.01 | 0.003-0.04 | 0 | 0-0.007 |
| Mel109 | 0.07 | 0.04-0.1 | 0.08 | 0.05-0.1 |
| Mel110 | 0.02 | 0.003-0.05 | 0.004 | 0.00008-0.02 |
| Mel111 | 0.08 | 0.04-0.1 | 0.04 | 0.01-0.07 |
| Mel112 | 0.006 | 0-0.03 | 0.003 | 0.00006-0.02 |
| Mel113 | 0.06 | 0.02-0.1 | 0.02 | 0.005-0.06 |
| Mel114 | 0.05 | 0.004-0.2 | 0 | 0-0.06 |
| Mel115 | 0.02 | 0.004-0.04 | 0.006 | 0.0005-0.02 |
| Mel116 | 0.1 | 0.05-0.3 | 0.2 | 0.002-0.07 |
| Mel117 | 0.009 | 0.001-0.03 | 0 | 0-0.01 |
|  |  |  |  |  |

Table S2. Best linear unbiased predictor (BLUP) values (represent the predicted deviation of each (maternal and paternal) social group from the mean paternity distance) and standard errors for each maternal (M) and paternal (P) social group extracted from the PD*_i_* model. Values represent the predicted deviation of each social group from the mean. Groups with missing data had no parentage assignments, therefore BLUPs were not estimated. Results are on the log-transformed scale with untransformed PD in meters.

| **Social group** | **BLUP_P_ (SE)** | **BLUP_M_ (SE)** |
| --- | --- | --- |
| Arthurs | 0.31 (0.29) | -0.27 (0.25) |
| Atcombe West | -0.09 (0.75) | 0.07 (0.62) |
| Atcombe Corner | 1.12 (0.53) | -0.92 (0.43) |
| Bamboo | 0.08 (0.56) | -0.07 (0.46) |
| Beech | 0.26 (0.26) | -0.17 (0.22) |
| Bungalow | -0.63 (0.55) | 0.52 (0.45) |
| Cedar | 0.35 (0.31) | -0.31 (0.26) |
| Cole Park | -0.88 (0.63) | 0.72 (0.52) |
| Colliers Wood | 0.03 (0.3) | 0.01 (0.28) |
| Convent | - | - |
| Dark Wood | - | -0.38(0.45) |
| Dingle | -0.73 (0.53) | 0.59 (0.43) |
| Field Farm | 0.55 (0.50) | -0.47 (0.42) |
| Gully | - | - |
| Hedge | -0.36 (0.35) | 0.30 (0.29) |
| Hogarths | - | - |
| Holly Wood | 0.41 (0.41) | 0.41 (0.41) |
| Honeywell | 0.65 (0.41) | -0.56 (0.34) |
| Inchbrook | 0.12 (0.42) | -0.14 (0.35) |
| Jacks Mirey | 1.16 (0.32) | -0.95 (0.27) |
| Kennel | -0.003 (0.30) | 0.036 (0.25) |
| Larch | 0.14 (0.29) | -0.10 (0.25) |
| Listers | -0.73 (0.59) | 0.59 (0.48) |
| Nettle | 0.64 (0.47) | -0.52 (0.39) |
| Old Oak | 0.38 (0.37) | -0.32 (0.31) |
| Park Mill | 0.11 (0.39) | -0.09 (0.33) |
| Peglars | 0.02 (0.37) | -0.02 (0.31) |
| Septic Tank | 0.56 (0.28) | -0.38 (0.24) |
| Thistle Wood Bank | - | - |
| Top Sett | -1.97 (0.32) | 1.60 (0.26) |
| West | 0.17 (0.34) | -0.09 (0.28) |
| Windsor Edge | 0.76 (0.33) | -0.60 (0.28) |
| Wood Farm | -0.42 (0.33) | 0.34 (0.27) |
| Wych Elm | -0.25 (0.32) | 0.19(0.26) |
| Yew | -0.55 (0.29) | 0.42 (0.25) |

Table S3. Reanalysis of PDi and EGPi using standardised body mass index (SMI) in place of body mass. Response variables were standardised into standard deviation units (SDU) prior to analysis. M and P denote maternal and paternal individuals, while MSG and PSG denote the corresponding maternal and paternal social groups.

|  | **Log(PD_i_)** | | | | **EGP_i_** | | | |
| --- | --- | --- | --- | --- | --- | --- | --- | --- |
|  | **Estimate (SE)** | **DF** | **F** | **P** | **Estimate (SE)** | **DF** | **F** | **P** |
| Intercept | 0.73 (0.16) | 1, 284.6 | 20.47 | **<0.001** | 0.74 (0.16) | 1, 297.7 | 21.01 | **<0.001** |
| Age_M_ | -0.009 (0.01) | 1, 539.9 | 0.55 | 0.46 | 0.01 (0.01) | 1, 543.3 | 0.64 | 0.42 |
| SMI_M_^†^ | 0.009 (0.01) | 1, 333.3 | 0.78 | 0.38 | 0.009 (0.01) | 1, 336.9 | 0.69 | 0.41 |
| Group_size_MSG_ | 0.007 (0.02) | 1, 461.4 | 0.19 | 0.66 | 0.008 (0.02) | 1, 446.9 | 0.20 | 0.66 |
| Sex_ratio_MSG_ ^‡^ | -0.71 (0.22) | 1, 533.4 | 10.28 | **<0.001** | -0.79 (0.22) | 1, 526.5 | 12.74 | **<0.001** |
| Age_P_ | 0.03 (0.02) | 1, 506.6 | 2.24 | 0.14 | 0.03 (0.02) | 1, 507.9 | 2.54 | 0.11 |
| SMI_P_^†^ | -0.02 (0.01) | 1, 247.7 | 1.15 | 0.29 | -0.02 (0.01) | 1, 249.9 | 1.11 | 0.29 |
| Group.Size_PSG_ | -0.02 (0.02) | 1, 538.2 | 0.69 | 0.41 | -0.02 (0.02) | 1, 532.4 | 0.68 | **0.04** |
| Sex_ratio_PSG_ ^‡^ | 0.42 (0.24) | 1, 539.6 | 3.06 | 0.08 | 0.50 (0.24) | 1, 537.1 | 4.29 | **<0.001** |

^†^mean body mass for parental individuals with multiple weight measurements within cub’s birth year

^‡^calculated as number of males divided by group size where group size is males plus females

Full models fitted for each response were y ~ μ + Age_M_ + SMI_M_ + Group_size_MSG_ + Sex_ratio_MSG_ + Age_P_ + SMI_P_ + Group_size_PSG_ + Sex_ratio_PSG_ + *M* + *P* + *MSG* + *PSG* + *Year* where italic font denotes random effects and y is either log(PD_i_) or EGP_i_

Table S4. Estimated (co)variance components (standard error) associated with random effects in mixed models of EGP*_i_* and log-transformed PD*_i_*, reanalysed using using standardised body mass index (SMI) in place of body mass. Statistical inference of random effects is by likelihood ratio test results (see main text for details). M and P denote maternal and paternal individuals, while MSG and PSG denote the corresponding maternal and paternal social groups.

|  |  | **log(PD*_i_*)** |  |  |  | **EGP*_i_*** |  |  |
| --- | --- | --- | --- | --- | --- | --- | --- | --- |
|  | **Variance (SE)** | **df** | **χ^2^_1_** | **P** | **Variance (SE)** | **df** | **χ^2^_1_** | **P** |
| **V_year_** | 0.06 (0.02) | 1 | 3.76 | **0.05** | 0.02 (0.01) | 1 | 3.20 | 0.07 |
| **V_M_^†^** | 0.25 (0.05) | 1 | 40.74 | **<0.001** | 0.26 (0.05) | 1 | 40.91 | **<0.001** |
| **V_P_^†^** | 0.31 (0.06) | 1 | 35.22 | **<0.001** | 0.31 (0.06) | 1 | 34.71 | **<0.001** |
| **V_MSG_^‡^** | 0.41 (0.15) | 1 | 20.64 | **<0.001** | 0.35 (0.13) | 1 | 19.92 | **<0.001** |
| **V_PSG_^‡^** | 0.60 (0.21) | 1 | 26.57 | **<0.001** | 0.54 (0.19) | 1 | 27.5 | **<0.001** |
| ***COV_MSG,PSG_*** | *-0.49 (0.17)* | *1* | *39.84* | **<0.001** | *-0.44 (0.15)* | *1* | *37.05* | **<0.001** |
| **V_R_** | 0.32 (0.04) | - | - | - | 0.32 (0.03) | - | - | - |

^†^ not significantly different from each other (logLRT, PD_i_: χ^2^_1_ = 0.22, p=0. 0.64; EGP_i_: χ^2^_1_ = 0.30, p=0.59)

^‡^ not significantly different from each other (logLRT, PD_i_: χ^2^_1_ = 3.73, p= 0.05; EGP_i_: χ^2^_1_ = 3.69, p=0.05)

Table S5. Repeatabilities (R) of variance components from reanalyses of EGP*_i_* and log-transformed PD*_i_*, reanalysed using standardised body mass index (SMI) in place of body mass. R calculated as variance component/sum of all variance components. Values for *COR_MSG,PSG_* are correlation coefficients. M and P denote maternal and paternal individuals, while MSG and PSG denote the corresponding maternal and paternal social groups.

|  |  | | **log(PD*_i_*)** | **EGP*_i_*** | |
| --- | --- | --- | --- | --- | --- |
|  | | **R (SE)** | | **R (SE)** | |
| **R_year_** | | 0.01(0.008) | | | 0.009 (0.008) |
| **R_M_^a^** | | 0.13 (0.04) | | | 0.14 (0.04) |
| **R_P_^a^** | | 0.16 (0.04) | | | 0.17 (0.04) |
| **R_MSG_^b^** | | 0.22 (0.05) | | | 0.20 (0.05) |
| **R_PSG_^b^** | | 0.31 (0.06) | | | 0.30 (0.06) |
| ***COR_MSG,PSG_*** | | *-0.99 (0.03)* | | | *-0.99 (0.03)* |
| **R_R_** | | 0.17 (0.04) | | | 0.18 (0.04) |

Table S6. Estimated fixed effect coefficients (standard error) and Wald F-tests from mixed models from reanalysis on log-PD_i_ and EGP_i_ using the 95% confidence pedigree, where only those parent assignments that met a 95% confidence threshold were included. Response variables were standardised into standard deviation units (SDU) prior to analysis. M and P denote maternal and paternal individuals, while MSG and PSG denote the corresponding maternal and paternal social groups.

|  | **Log(PD_i_)** | | | | **EGP_i_** | | | |
| --- | --- | --- | --- | --- | --- | --- | --- | --- |
|  | **Estimate (SE)** | **DF** | **F** | **P** | **Estimate (SE)** | **DF** | **F** | **P** |
| Intercept | 0.62 (0.23) | 1, 94.3 | 7.22 | **<0.01** | 0.64 (0.23) | 1, 92.7 | 7.51 | **<0.01** |
| Age_M_ | -0.01 (0.008) | 1, 115.9 | 0.01 | 0.91 | 0.002 (0.007) | 1, 115.5 | 0.09 | 0.76 |
| Body mass_M_^†^ | -0.04 (0.026) | 1, 219.1 | 2.01 | 0.16 | -0.04 (0.03) | 1, 220.0 | 2.09 | 0.15 |
| Group_size_MSG_ | 0.12 (0.01) | 1, 96.0 | 127.59 | **<0.001** | 0.13 (0.01) | 1, 88.3 | 185.62 | **<0.001** |
| Sex_ratio_MSG_ ^‡^ | -3.29 (0.14) | 1, 142.5 | 535.52 | **<0.001** | -3.65 (0.13) | 1, 135.1 | 805.31 | **<0.001** |
| Age_P_ | 0.03 (0.008) | 1, 112.0 | 19.16 | **<0.001** | 0.03 (0.007) | 1, 112.9 | 18.67 | **<0.001** |
| Body mass_P_^†^ | -0.02 (0.03) | 1, 156.8 | 0.71 | 0.40 | -0.02 (0.03) | 1, 160.7 | 0.66 | 0.42 |
| Group.Size_PSG_ | -0.08 (0.01) | 1, 97.0 | 28.90 | **<0.001** | -0.09 (0.01) | 1, 89.4 | 52.63 | **<0.001** |
| Sex_ratio_PSG_ ^‡^ | 2.84 (0.17) | 1, 160.2 | 287.70 | **<0.001** | 3.22 (0.15) | 1, 149.8 | 446.25 | **<0.001** |

^†^mean body mass for parental individuals with multiple weight measurements within cub’s birth year

^‡^calculated as number of males divided by group size where group size is males plus females

Full models fitted for each response were y ~ μ + Age_M_ + Body_Mass_M_ + Group_size_MSG_ + Sex_ratio_MSG_ + Age_P_ + Body_Mass_P_ + Group_size_PSG_ + Sex_ratio_PSG_ + *M* + *P* + *MSG* + *PSG* + *Year* where italic font denotes random effects and y is either log(PD_i_) or EGP_i_

Table S7. Estimated (co)variance components (standard error) associated with random effects in mixed models of EGP*_i_* and log-transformed PD*_i_*, reanalysed using 95% confidence pedigree*_­_*. Statistical inference of random effects is by likelihood ratio test results (see main text for details). M and P denote maternal and paternal individuals, while MSG and PSG denote the corresponding maternal and paternal social groups.

|  |  | **log(PD*_i_*)** |  |  |  | **EGP*_i_*** |  |  |
| --- | --- | --- | --- | --- | --- | --- | --- | --- |
|  | **Variance (SE)** | **df** | **χ^2^_1_** | **P** | **Variance (SE)** | **df** | **χ^2^_1_** | **P** |
| **V_year_** | 0.04 (0.02) | 1 | 45.88 | **<0.001** | 0.04 (0.02) | 1 | 60.89 | **<0.001** |
| **V_M_^†^** | 1.86 (0.23) | 1 | 115.12 | **<0.001** | 1.94 (0.24) | 1 | 131.15 | **<0.001** |
| **V_P_^†^** | 1.80 (0.25) | 1 | 98.34 | **<0.001** | 1.93 (0.26) | 1 | 114.94 | **<0.001** |
| **V_MSG_**^‡^ | 2.21 (0.71) | 1 | 41.93 | **<0.001** | 2.13 (0.68) | 1 | 55.91 | **<0.001** |
| **V_PSG_**^‡^ | 2.22 (0.71) | 1 | 197.85 | **<0.001** | 2.16 (0.69) | 1 | 80.31 | **<0.001** |
| ***COV_MSG,PSG_*** | *-2.04 (0.66)* | *1* | *37.27* | **<0.001** | *-1.96 (0.64)* | *1* | *35.58* | **<0.001** |
| **V_R_** | 0.005 (0.0008) | - | - | - | 0.004 (0.0006) | - | - | - |

**^†^**not significantly different from each other (logLRT, PD_i_: χ^2^_1_ = 0.038, p=0.85; EGP_i_: χ^2^_1_ = 0.002, p=0.96)

^‡^ not significantly different from each other (logLRT, PD_i_: χ^2^_1_ = 0, p= 1; EGP_i_: χ^2^_1_ = 0.006, p=0.94)

Table S8. Repeatabilities (R) of variance components from reanalyses of EGP*_i_* and log-transformed PD*_i_*, reanalysed using 95% confidence pedigree*_­_*. R calculated as variance component/sum of all variance components. Values for *COV_MSG,PSG_* are correlation coefficients. M and P denote maternal and paternal individuals, while MSG and PSG denote the corresponding maternal and paternal social groups.

|  |  | | **log(PD*_i_*)** | **EGP*_i_*** | |
| --- | --- | --- | --- | --- | --- |
|  | | **R (SE)** | | **R (SE)** | |
| **R_year_** | | 0.005(0.002) | | | 0.004 (0.002) |
| **R_M_^a^** | | 0.23 (0.04) | | | 0.24 (0.04) |
| **R_P_^a^** | | 0.22 (0.04) | | | 0.24 (0.04) |
| **R_MSG_^b^** | | 0.27 (0.05) | | | 0.26 (0.05) |
| **R_PSG_^b^** | | 0.27 (0.05) | | | 0.26 (0.05) |
| ***COR_MSG,PSG_*** | | *-0.92 (0.05)* | | | *-0.92 (0.05)* |
| **R_R_** | | 0.0007 (0.0002) | | | 0.0005 (0.05) |

Table S9. Posterior mean (credible intervals) estimates of unsampled males and females per cohort estimated in MasterBayes simultaneously with parentage and paternity distance. Values for unsampled males represent population-level estimates, while number of unsampled females was estimated per social group.

| **Year** | **Unsampled males** | **Unsampled females** |
| --- | --- | --- |
| 1986 | 11.827 (0.407-43.445) | 1.2651 (0.316-3.474) |
| 1987 | 35.622 (6.093-89.752) | 0.819 (0.1945-1.958) |
| 1988 | 26.1864 (0.805-96.173) | 0.975 (0.122-2.801) |
| 1989 | 6.401 (0.250-21.541) | 0.548 (0.044-1.626) |
| 1990 | 10.764 (0.676-32.665) | 0.803 (0.054-2.465) |
| 1991 | 16.404 (1.693-44.149) | 2.314 (0.960-4.439) |
| 1992 | 37.147 (6.335-90.084) | 0.380 (0.011-1.269) |
| 1993 | 20.403 (0.6008-68.087) | 0.843 (0.123-2.136) |
| 1994 | 12.696 (0.680-39.903) | 0.359 (0.009-1.341) |
| 1995 | 40.303 (5.744-102.097) | 6.283 (2.062-12.629) |
| 1996 | 239.383 (32.810-812.610) | 2.370 (0.091-7.561) |
| 1997 | 16.930 (2.090-43.140) | 0.980 (0.100-2.640) |
| 1998 | 47.200 (17.15-89.200 | 0.540 (0.110-1.250) |
| 1999 | 35.000 (10.390-71.470) | 0.650 (0.160-1.400) |
| 2000 | 35.081 (12.290-68.310) | 0.799 (0.239-1.76) |
| 2001 | 28.868 (12.280-50.640) | 0.604 (0.226-1.174) |
| 2002 | 55.9474 (12.230-132.450) | 0.428 (0.010-1.610) |
| 2003 | 24.937 (5.672-55.067) | 0.517 (0.066-1.312) |
| 2004 | 20.150 (2.961-49.365) | 0.705 (0.142-1.695) |
| 2005 | 10.192 (1.035-26.744) | 0.919 (0.313-1.850) |
| 2006 | 5.129 (0.208-17.236) | 2.653 (1.141-4.951) |
| 2007 | 6.859 (0.389-20.929) | 1.363 (0.403 -2.915) |
| 2008 | 18.010 (4.195-39.820) | 1.896 (0.661-3.836) |
| 2009 | 16.416 (5.726-31.224) | 0.702 (0.210-1.462) |
| 2010 | 18.812 (0.970-57.216) | 2.353 (0.482-6.379) |
| 2011 | 6.698 (0.168-23.674) | 1.3234 (0.308-2.703) |
| 2012 | 49.145 (13.680-105.520) | 0.739 (0.153-1.795) |
| 2013 | 50.206 (21.790-88.780) | 0.614 (0.167-1.338) |
| 2014 | 111.922 (49.660-217.92) | 2.225 (1.000-4.137) |

Figure S1. Inferred pedigree structure for 29 cohorts showing maternal assignments in red, paternal in blue and individuals as dots. Reconstructed pedigree has a maximum depth of six generations and contains 579 maternal-cub and 596 paternal-cub links, 186 full sibships, 452 maternal half-sibs, and 927 paternal half sibs.


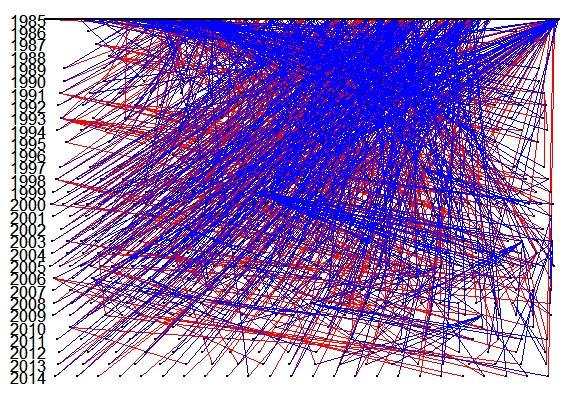


Figure S2. Histogram of model residuals for binary EGP_i_ (0/1) run in ASReml 3.0 with a Gaussian error structure.


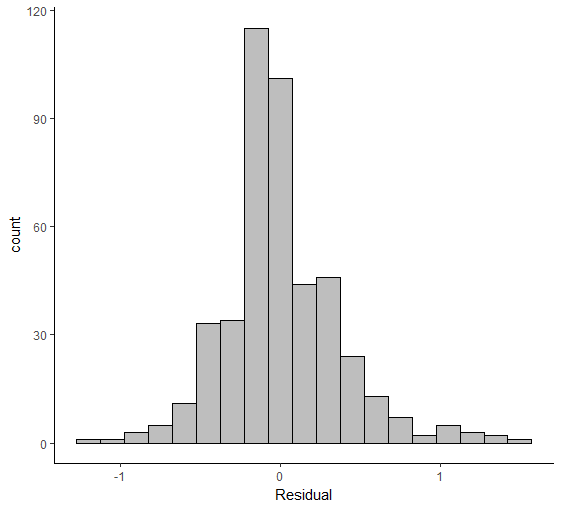

Supplement: Supplementary Data [file ary164_suppl_supplementary-data.docx]
